# dTAPL, A Method That Expedites the Discovery of Proteins Associated With Specific Genomic Loci in Plants

**DOI:** 10.1111/pbi.70336

**Published:** 2025-08-20

**Authors:** Fan Zhang, Tengfei Zhu, Conggui Peng, Zhen Zhuang, Yaping Tang, Zhi Yang, Shengbao Yang, Liyu Huang, Xu Wang

**Affiliations:** ^1^ Shandong Laboratory of Advanced Agricultural Sciences in Weifang Peking University Institute of Advanced Agricultural Sciences Weifang Shandong China; ^2^ College of Life Sciences Shandong Agricultural University Tai'an China; ^3^ Key Laboratory of Biology and Germplasm Innovation of Perennial Rice From Ministry of Agriculture and Rural Affairs, School of Agriculture Yunnan University Kunming Yunnan China; ^4^ Institute of Horticulture Crops Xinjiang Academy of Agricultural Sciences Urumqi Xinjiang China

**Keywords:** CRISPR/dCas9, genomic locus‐specific binding proteins, TurboID

Control of gene transcription is fundamental to cell function which is established through the dynamically changed occupancy of proteins at regulatory elements. Defining the in vivo interactome of proteins and genomic loci is key to deciphering the rules that govern gene transcription. Chromatin immunoprecipitation (ChIP) is the most widely used method for capturing the protein‐chromatin/DNA complexes, and it can be coupled with sensitive mass spectrometry (MS) for protein identification. ChIP relies on antibodies that recognise specific proteins, not DNA; thus, the data acquired by ChIP‐MS is not DNA site‐specific. Therefore, purifying native DNA site‐specific regions and identifying proteins bound with them remain ongoing challenges. The advent of cutting‐edge technologies including CRISPR/Cas9 genome editing and protein Proximity Labeling (PL) has provided powerful tools to tackle this challenge. CRISPR/Cas9 implements the CRISPR RNA‐guided DNA cleavage by Cas9 nuclease (Jinek et al. 2012). Noteworthily, a nuclease‐deficient mutant of Cas9 (dCas9) has been exploited as a versatile tool for precise genome targeting. When applied in plant cells, dCas9 could target diverse types of DNA elements in the presence of specific guide RNAs, including promoters (Chen et al. 2024), enhancers (Zhang et al. 2025), protein‐coding genes (Papikian et al. 2019), transposable elements (Papikian et al. 2019), telomere regions (Dreissig et al. 2017) and centromeres (Potlapalli et al. 2024), suggesting the potential for broad applications of dCas9 towards elucidating interactions of important DNA elements and the factors bound with them. PL uses engineered enzymes to covalently tag the neighbouring proteins with biotin or biotin derivatives in minutes (Qin et al. 2021). Techniques based on the above methods have been recently developed in animal cells to map protein‐genomic loci interactions, such as CasID, CAPLOCUS, GLoPro and C‐BERST (Gauchier et al. 2020).

However, hitherto, no such in vivo technique is available for plant research. Here, we describe a method called *dCas9‐TurboID Assisted Proximity Labeling of proteins in specific genomic context* (dTAPL), in which CRISPR/dCas9‐based genome targeting and TurboID‐catalysed PL are assembled to profile proteomic composition at specific genomic loci in plant cells. To construct the dTAPL plasmid, the *dCas9* was fused in‐frame with the coding sequence of TurboID to allow the production of recombinant dCas9‐TurboID proteins. The Csy4‐processed polycistronic guide RNAs (gRNAs) system was also adopted to enable the simultaneous targeting of multiple genomic loci (Cermak et al. 2017). We then introduced the dTAPL plasmids into tobacco leaves via agroinfiltration with various cell densities of agrobacterium culture and different harvest times post infiltration. The optimal protein expression was observed when the concentration of agrobacterium at OD_600_ = 0.4 was used, and samples were collected in 2 days afterwards (Figure S2). Next, different concentrations of biotin were tested, and a steep increase in labeling efficiency occurred when 50 μM biotin was used (Figure 1b). Moreover, the expression of dTAPL plasmids was confirmed in pepper seedlings ( *C. annuum* ) by agroinfiltration (Figure S3), suggesting potential applications of dTAPL in other dicots. As the dTAPL plasmid was a binary vector, besides being used in transient expression assays, it could also be used for generating stable transgenic plants.

**FIGURE 1:**
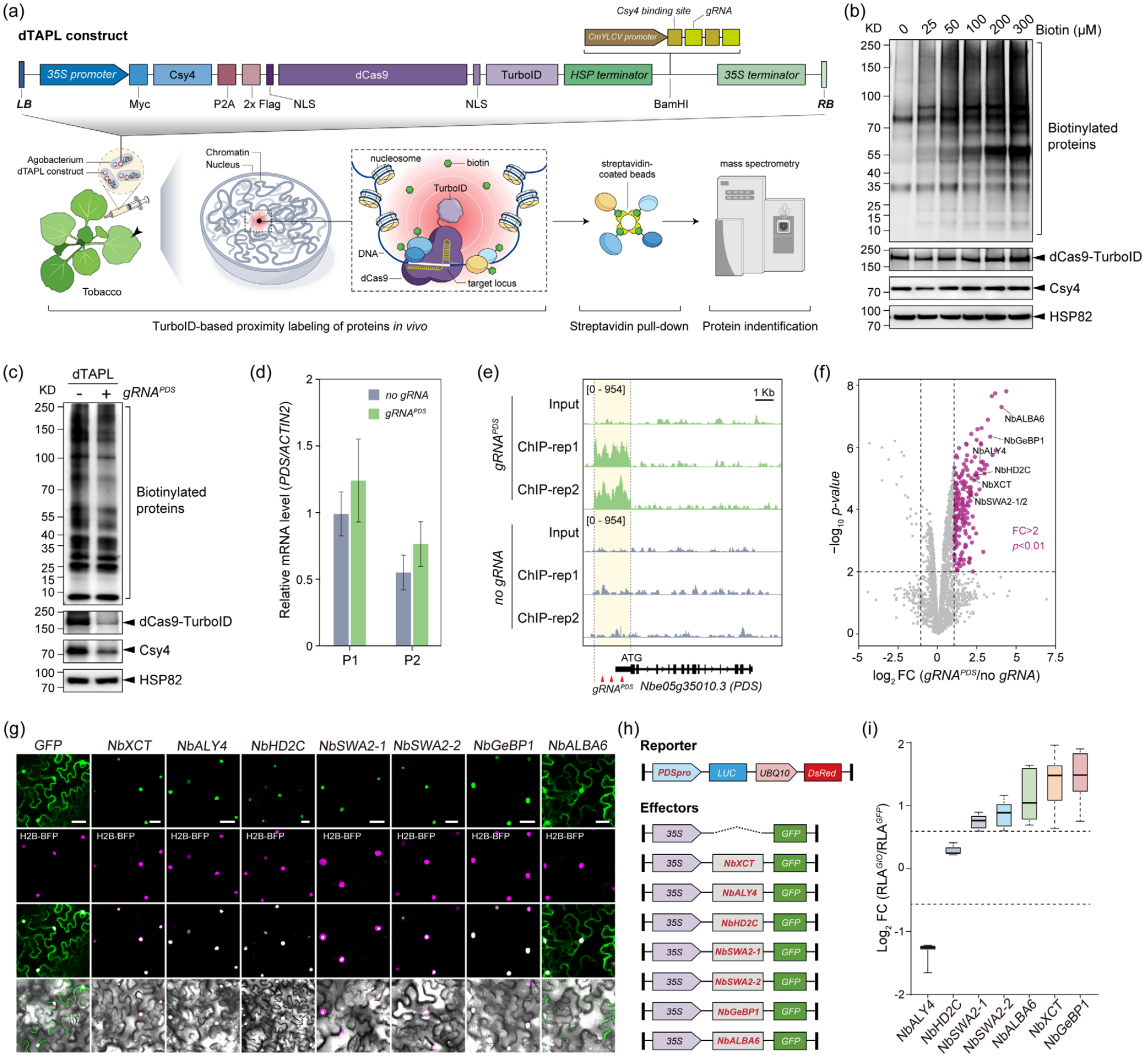
Rationale of dTAPL and its application in identifying candidate factors bound with *PDS* promoter. (a) Structure of dTAPL plasmid and the experimental workflow of dTAPL method used in this study. (b) Immunoblots showing the expression levels of indicated proteins in tobacco leaves for determining the optimal biotin concentrations used for proximity labeling. (c) Immunoblots showing the expression of proteins indicated. (d) Real‐time qPCR analysis of *PDS* mRNA level in the tobacco leaves where dTAPL plasmids expressed with or without gRNA targeting to *PDS* promoter (*gRNA* ^*PDS*^ or no *gRNA*). Two pairs of primers (P1 and P2) were used to detect *PDS* expressions. (e) ChIP‐seq results showing the binding peaks of dCas9‐TurboID at *PDS* promoter in the presence of *gRNA* ^*PDS*^. (f) Volcano plot showing the dTAPL proteomics in tobacco leaves. Significantly enriched proteins (*p* < 0.01, FC > 2) were denoted by magenta closed circles. (g) Subcellular localizations of selected candidate proteins. Histone H2B tagged with Blue Fluorescence Protein (H2B‐BFP) was used as the nuclear marker. Scale bar = 50 μm. (h) Structures of LUC reporter and effector constructs. (i) LUC reporter assays showing the effects of selected proteins on regulating *PDS* promoter activities. Relative LUC activity (RLA) for each effector was calculated by normalising the LUC intensity with DsRed intensity of the same region on the image. GOI, gene of interest.

For proof‐of‐concept testing of dTAPL, we selected the *Phytoene Desaturase* (*PDS*) gene for analysis. *PDS* encodes a key enzyme in carotenoid biosynthesis and knockout of this gene leads to an albino phenotype in many plant species (Qin et al. 2007). However, the regulatory mechanisms of *PDS* transcription are unknown. Given tethering dCas9‐TurboID to chromatin might interfere with the endogenous protein‐genome interactions, the gRNAs were designed to avoid the predicted TF binding motifs in the *PDS* promoter region. We first confirmed the expressions of dCas9‐TurboID and Csy4 proteins in tobacco leaves (Figure 1c); then compared the mRNA levels of *PDS* between the samples expressing gRNAs targeting the promoter of *PDS* ((+) *gRNA* ^*PDS*^) or no gRNAs ((−) *gRNA* ^*PDS*^) as the negative control. The results showed that the *PDS* mRNA level remained unchanged between the two samples (Figure 1d), suggesting that the local interactome between the endogenous proteins and the *PDS* loci might not be influenced by dCas9‐TurboID targeting. Then, chromatin immunoprecipitation followed by sequencing (ChIP‐seq) assays were performed with the same samples used for detecting *PDS* expressions. The ChIP‐seq results plainly revealed that dCas9‐TurboID proteins bound with the *PDS* promoter only in the presence of *gRNA* ^*PDS*^ (Figure 1e), indicating that gRNAs effectually directed the dCas9‐TurboID to the target loci. It should also be noted that off‐target peaks have been seen in ChIP‐seq results (Figure S4); this phenomenon may vary depending on the locus, the number of gRNAs, or the expression level of both gRNAs and dCas9 proteins. Therefore, optimization might be needed when using dTAPL for different experimental purposes. Lastly, the biotinylated proteins were purified through streptavidin selection and analysed by MS. Through label‐free quantification method to measure enrichment in the “*gRNA* ^*PDS*^” sample relative to protein amounts in the “*no gRNAs*” sample (*p* < 0.01 and Fold Change (FC) ≥ 2.0), 200 candidate proteins were identified, of which 91 were predicted as nuclear‐localised proteins (Figure 1f; Table S1). We selected seven candidates for functional analysis, including transcription factor (NbGeBP1), transcription co‐regulator (NbALY4), chromatin remodelers (NbHD2C and NbXCT) and RNA‐binding proteins (NbALBA6, NbSWA2‐1 and NbSWA2‐2). The results of the subcellular localization assay indicated that six out of seven selected candidates were nuclear proteins (NbXCT, NbALY4, NbHD2C, NbSWA2‐1, NbSWA2‐2 and NbGbBP1); only NbALBA6 distributed both in the nucleus and cytoplasm (Figure 1g). We next examined the activities of these proteins in regulating the expression of luciferase (LUC) reporter under the control of the *PDS* promoter. By quantifying the relative activities of LUC reporters with different effectors (Figure 1h,i), we found that the *PDS* promoter could be markedly repressed by NbALY4 or stimulated by NbXCT, NbSWA2‐1, NbSWA2‐2 and NbGbBP1 (Figure 1h,i; Figure S5), suggesting that these candidates might be potential regulators of *PDS* expression in vivo. To further validate the results obtained by dTALP*‐gRNA* ^*PDS*^, two experiments were performed: First, we transiently expressed four candidate genes (*NtALY4*, *NtXCT*, *NtGeBP1* and *NtALBA6*) individually in tobacco leaves, which were then used for ChIP‐qPCR analysis. The results showed that NtALY4‐GFP, NtGeBP1‐GFP and NtALBA6‐GFP significantly enriched at the chromatin region of the *PDS* promoter compared to the results of the control sample expressing free GFP (Figure S6a), indicating that those factors identified by dTAPL indeed associate with the *PDS* promoter in vivo. Second, *dTALP‐gRNA* ^*PDS*^ or *dTAPL* DNA construct was co‐expressed with the candidate genes mentioned above in tobacco leaves, which were then fed with biotin to initiate proximity labeling. The immunoblots evidently showed that biotinylated proteins were more abundant in the presence of *gRNA* ^*PDS*^ (Figure S6b), suggesting that those factors are in the proximity of the dCas9‐TurboID/*gRNA* ^*PDS*^ complex. Further protein interaction network analysis and functional validation of a selected ribosome biosynthesis cofactor, NbSWA2‐1 (Figure S7), strongly argue that this rRNA‐related factor might serve as a *bona fide* regulator of *PDS* expression in tobacco. In summary, dTAPL promises to augment the limited options of in vivo tools in plants to identify candidate factors that are associated with functionally important *cis*‐elements and chromatin regions, thereby expediting the annotation of these factors and their roles.

## Author Contributions

X.W., L.H., S.Y. and F.Z. designed the experiments. F.Z., T.Z., C.P. and Y.T. performed all the experiments. Z.Z. analysed the ChIP‐seq and MS data. X.W. wrote the manuscript. All the authors read and edited the manuscript.

## Supporting information


Figure S1–S7.
Appendix S1.

Table S1–S2.

## Data Availability

The data that support the findings of this study are available on request from the corresponding author. The data are not publicly available due to privacy or ethical restrictions.
