# Supplementary material for: dTAPL, A Method That Expedites the Discovery of Proteins Associated With Specific Genomic Loci in Plants
Source: Plant Biotechnol J. 2025 Aug 20;23(12):5677–9. doi: 10.1111/pbi.70336 (PMC12665084; doi:10.1111/pbi.70336)
Supplement: Supplementary file 1 — Figure S1–S7. Appendix S1. [file PBI-23-5677-s001.pdf]

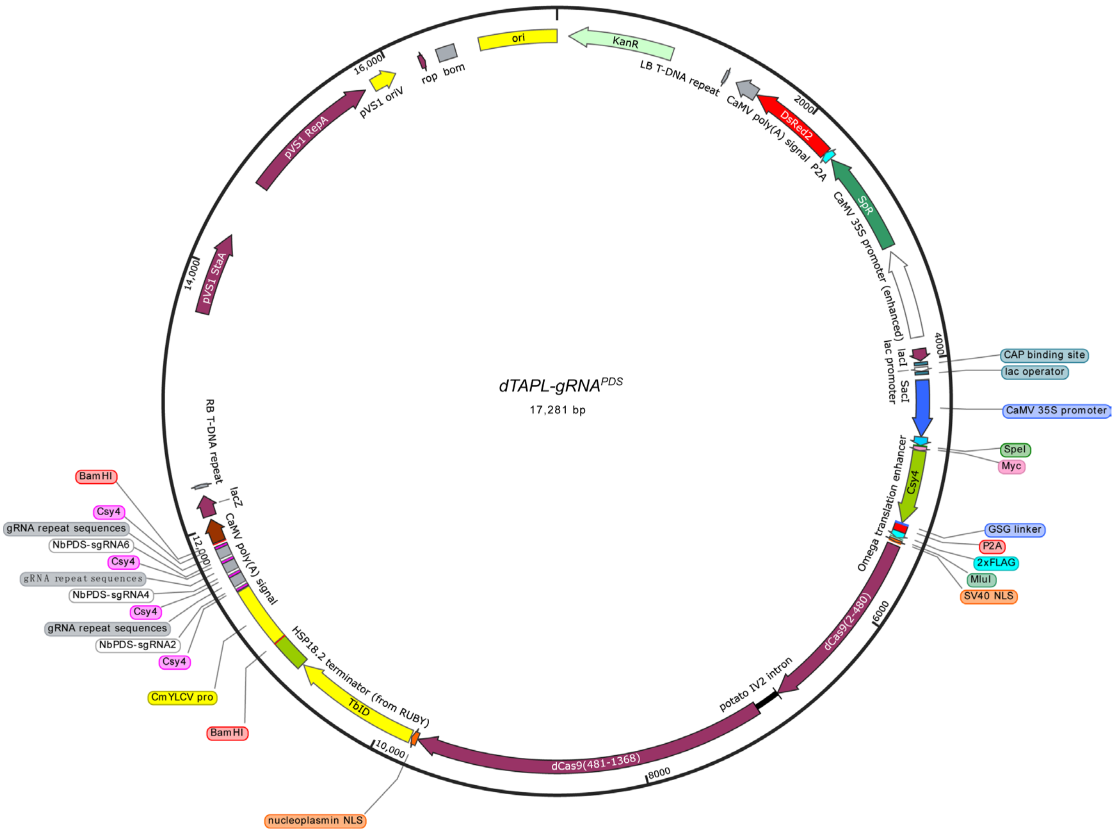

Full sequence of dTAPL plasmid:

```
LOCUS       Exported              16416 bp DNA     circular SYN 17-MAY-2024
SOURCE      synthetic DNA construct
REFERENCE   1  (bases 1 to 16416)
FEATURES             Location/Qualifiers
     source          1..16416
                     /mol_type="other DNA"
                     /organism="synthetic DNA construct"
     CDS             complement(87..881)
                     /codon_start=1
                     /label=Kan
                     /translation="MAKMRISPELKKLIEKYRCVKDTEGMSPAKVYKLVGENENLYLKM
                     TDSRYKGTTYDVEREKDMMLWLEGKLPVPKVLHFERHDGWSNLLMSEADGVLCSEEYED
                     EQSPEKIIELYAECIRLFHSIDISDCPYTNSLDSRLAELDYLLNNDLADVDCENWEEDT
                     PFKDPRELYDFLKTEKPEEELVFSHGDLGDSNIFVKDGKVSGFIDLGRSGRADKWYDIA
                     FCVRSIREDIGEEQYVELFFDLLGIKPDWEKIKYYILLDELF"
     misc_feature    1306..1330
                     /label=LB T-DNA repeat
     primer_bind     1348..1369
                     /label=#100-seqR
                     /note="sequence: ACTTAATAACACATTGCGGACG"
```

```
polyA_signal    complement(1408..1582)
                /label=CaMV poly(A) signal
CDS             complement(1589..2266)
                /codon_start=1
                /label=DsRed2
                /translation="MASSENVITEFMRFKVRMEGTVNGHEFEIEGEGEGRPYEGHNTVK
                LKVTKGGPLPFAWDILSPQFQYGSKVYVKHPADIPDYKKLSFPEGFKWERVMNFEDGGV
                ATVTQDSSLQDGCFIYKVKFIGVNFPSDGPVMQKKTMGWEASTERLYPRDGVLKGETHK
                ALKLKDGGHYLVEFKSIYMAKKPVQLPGYYYVDAKLDITSHNEDYTIVEQYERTEGRHH
                LFL"
CDS             complement(2267..2323)
                /codon_start=1
                /label=P2A
                /translation="ATNFSLLKQAGDVEENPGP"
CDS             complement(2333..3121)
                /codon_start=1
                /label=SpR
                /translation="MGEAVIAEVSTQLSEVVGVIERHLEPTLLAVHLYGSAVDGGLKPH
                SDIDLLVTVTVRLDETTRRALINDLLETSASPGESEILRAVEVTIVVHDDIIPWRYPAK
                RELQFGEWQRNDILAGIFEPATIDIDLAILLTKAREHSVALVGPAAEELFDPVPEQDLF
                EALNETLTLWNSPPDWAGDERNVVLTLSRIWYSAVTGKIAPKDVAADWAMERLPAQYQP
                VILEARQAYLGQEEDRLASRADQLEEFVHYVKGEITKVVGK"
promoter        complement(3160..3836)
                /label=CaMV 35S promoter (enhanced)
protein_bind    4027..4048
                /label=CAP binding site
promoter        4063..4093
                /label=lac promoter
protein_bind    4097..4119
                /label=lac operator
promoter        4162..4588
                /label=CaMV 35S promoter
misc_feature    4589..4653
                /label=Omega translation enhancer
CDS             4660..4692
                /codon_start=1
                /product="Myc (human c-Myc oncogene) epitope tag"
                /translation="MEQKLISEEDL"
CDS             4693..5253
                /codon_start=1
                /label=Csy4
                /translation="MDHYLDIRLRPDPEFPPAQLMSVLFGKLHQALVAQGGDRIGVSFP
                DLDESRSRLGERLRIHASADDLRALLARPWLEGLRDHLQFGEPAVVPHPTPYRQVSRVQ
                AKSNPERLRRRLMRRHDLSEEEARKRIPDTVARALDLPFVTLRSQSTGQHFRLFIRHGP
```

LQVTAEEGGFTCYGLSKGGFVPWF"

misc_feature   5254..5262

/label=GSG linker

/note="/Motif=GGAAGCGGC"

2A_signal   5263..5319

/label=P2A

CDS   5320..5370

/codon_start=1

/label=2xFLAG

/translation="MDYKDDDDKDYKDDDDK"

CDS   5383..5403

/codon_start=1

/product="nuclear localization signal of SV40 large T antigen"

/label=SV40 NLS

/translation="PKKKRKV"

CDS   5428..6864

/codon_start=1

/label=dCas9(2-480)

/translation="DKKYSIGLAIGTNSVGWAVITDEYKVPSKKFKVLGNTDRHSIKKN
LIGALLFDSGETAEATRLKRTARRRYTRRKNRICYLQEIFSNEMAKVDDSFFHRLEESF
LVEEDKKHERHPIFGNIVDEVAYHEKYPTIYHLRKKLVDSTDKADLRLIYLALAHMIKF
RGHFLIEGDLNPDNSDVDKLFIQLVQTYNQLFEENPINASGVDAKAILSARLSKSRRLE
NLIAQLPGEKKNGLFGNLIALSLGLTPNFKSNFDLAEDAKLQLSKDTYDDDLDNLLAQI
GDQYADLFLAAKNLSDAILLSDILRVNTEITKAPLSASMIKRYDEHHQDLTLLKALVRQ
QLPEKYKEIFFDQSKNGYAGYIDGGASQEEFYKFIKPILEKMDGTEELLVKLNREDLLR
KQRTFDNGSIPHQIHLGELHAILRRQEDFYPFLKDNREKIEKILTFRIPYYVGPLARGN
SRFAWMTRKSEETITPWNFEE"

intron   6865..7053

/label=potato IV2 intron

CDS   7054..9717

/codon_start=1

/label=dCas9(481-1368)

/translation="VVDKGASAQSFIERMTNFDKNLPNEKVLPKHSLLYEYFTVYNELT
KVKYVTEGMRKPAFLSGEQKKAIVDLLFKTNRKVTVKQLKEDYFKKIECFDSVEISGVE
DRFNASLGTYHDLLKIIKDKDFLDNEENEDILEDIVLTLTLFEDREMIEERLKTYAHLF
DDKVMKQLKRRRYTGWGRLSRKLINGIRDKQSGKTILDFLKSDGFANRNFMQLIHDDSL
TFKEDIQKAQVSGQGDSLHEHIANLAGSPAIKKGILQTVKVVDELVKVMGRHKPENIVI
EMARENQTTQKGQKNSRERMKRIEEGIKELGSQILKEHPVENTQLQNEKLYLYYLQNGR
DMYVDQELDINRLSDYDVDAIVPQSFLKDDSIDNKVLTRSDKNRGKSDNVPSEEVVKKM
KNYWRQLLNAKLITQRKFDNLTKAERGGLSELDKAGFIKRQLVETRQITKHVAQILDSR
MNTKYDENDKLIREVKVITLKSKLVSDFRKDFQFYKVREINNYHHAHDAYLNAVVGTAL
IKKYPKLESEFVYGDYKVYDVRKMIAKSEQEIGKATAKYFFYSNIMNFFKTEITLANGE
IRKRPLIETNGETGEIVWDKGRDFATVRKVLSMPQVNIVKKTEVQTGGFSKESILPKRN

```
                     SDKLIARKKDWDPKKYGGFDSPTVAYSVLVVAKVEKGKSKKLKSVKELLGITIMERSSF
                     EKNPIDFLEAKGYKEVKKDLIIKLPKYSLFELENGRKRMLASAGELQKGNELALPSKYV
                     NFLYLASHYEKLKGSPEDNEQKQLFVEQHKHYLDEIIEQISEFSKRVILADANLDKVLS
                     AYNKHRDKPIREQAENIIHLFTLTNLGAPAAFKYFDTTIDRKRYTSTKEVLDATLIHQS
                     ITGLYETRIDLSQLGGD"
CDS                  9718..9765
                     /codon_start=1
                     /label=nucleoplasmin NLS
                     /translation="KRPAATKKAGQAKKKK"
CDS                  9772..10746
                     /codon_start=1
                     /label=TurboID
                     /translation="GGSGSKDNTVPLKLIALLANGEFHSGEQLGETLGMSRAAINKHIQ
                     TLRDWGVDVFTVPGKGYSLPEPIPLLNAKQILGQLDGGSVAVLPVVDSTNQYLLDRIGE
                     LKSGDACIAEYQQAGRGSRGRKWFSPFGANLYLSMFWRLKRGPAAIGLGPVIGIVMAEA
                     LRKLGADKVRVKWPNDLYLQDRKLAGILVELAGITGDAAQIVIGAGINVAMRRVEESVV
                     NQGWITLQEAGINLDRNTLAATLIRELRAALELFEQEGLAPYLPRWEKLDNFINRPVKL
                     IIGDKEIFGISRGIDKQGALLLEQDGVIKPWMGGEISLRSAEK"
terminator           10758..11007
                     /label=HSP18.2 terminator (from RUBY)
terminator           11018..11192
                     /label=CaMV poly(A) signal
                     /label=RB T-DNA repeat
CDS                  12767..13396
                     /codon_start=1
                     /label=pVS1 StaA
                     /translation="MKVIAVLNQKGGSGKTTIATHLARALQLAGADVLLVDSDPQGSAR
                     DWAAVREDQPLTVVGIDRPTIDRDVKAIGRRDFVVIDGAPQAADLAVSAIKAADFVLIP
                     VQPSPYDIWATADLVELVKQRIEVTDGRLQAAFVVSRAIKGTRIGGEVAEALAGYELPI
                     LESRITQRVSYPGTAAAGTTVLESEPEGDAAREVQALAAEIKSKLI"
CDS                  13825..14898
                     /codon_start=1
                     /label=pVS1 RepA
                     /translation="VSGRKPSGPVQIGAALGDDLVEKLKAAQAAQRQRIEAEARPGESW
                     QAAADRIRKESRQPPAAGAPSIRKPPKGDEQPDFFVPMLYDVGTRDSRSIMDVAVFRLS
                     KRDRRAGEVIRYELPDGHVEVSAGPAGMASVWDYDLVLMAVSHLTESMNRYREGKGDKP
                     GRVFRPHVADVLKFCRRADGGKQKDDLVETCIRLNTTHVAMQRTKKAKNGRLVTVSEGE
                     ALISRYKIVKSETGRPEYIEIELADWMYREITEGKNPDVLTVHPDYFLIDPGIGRFLYR
                     LARRAAGKAEARWLFKTIYERSGSAGEFKKFCFTVRKLIGSNDLPEYDLKEEAGQAGPI
                     LVMRYRNLIEGEASAGS"
rep_origin           14964..15158
                     /label=pVS1 oriV
rep_origin           15828..16416
                     /label=ori
```

ORIGIN

```
        1 gaagatcctt tgatcttttc tacggggtct gacgctcagt ggaacgaaaa ctcacgttaa
       61 gggattttgg tcatgcattc taggtactaa aacaattcat ccagtaaaat ataatatttt
      121 attttctccc aatcaggctt gatccccagt aagtcaaaaa atagctcgac atactgttct
      181 tccccgatat cctccctgat cgaccggacg cagaaggcaa tgtcatacca cttgtccgcc
      241 ctgccgcttc tcccaagatc aataaagcca cttactttgc catctttcac aaagatgttg
      301 ctgtctccca ggtcgccgtg ggaaaagaca agttcctctt cgggcttttc cgtctttaaa
      361 aaatcataca gctcgcgcgg atctttaaat ggagtgtctt cttcccagtt ttcgcaatcc
      421 acatcggcca gatcgttatt cagtaagtaa tccaattcgg ctaagcggct gtctaagcta
      481 ttcgtatagg gacaatccga tatgtcgatg gagtgaaaga gcctgatgca ctccgcatac
      541 agctcgataa tcttttcagg gctttgttca tcttcatact cttccgagca aaggacgcca
      601 tcggcctcac tcatgagcag attgctccag ccatcatgcc gttcaaagtg caggaccttt
      661 ggaacaggca gctttccttc cagccatagc atcatgtcct tttcccgttc cacatcatag
      721 gtggtccctt tataccggct gtccgtcatt tttaaatata ggttttcatt ttctcccacc
      781 agcttatata ccttagcagg agacattcct tccgtatctt ttacgcagcg gtatttttcg
      841 atcagttttt tcaattccgg tgatattctc attttagcca tttattattt ccttcctctt
      901 ttctacagta tttaaagata ccccaagaag ctaattataa caagacgaac tccaattcac
      961 tgttccttgc attctaaaac cttaaatacc agaaaacagc tttttcaaag ttgttttcaa
     1021 agttggcgta taacatagta tcgacggagc cgattttgaa accgcggtga tcacaggcag
     1081 caacgctctg tcatcgttac aatcaacatg ctaccctccg cgagatcatc cgtgtttcaa
     1141 acccggcagc ttagttgccg ttcttccgaa tagcatcggt aacatgagca aagtctgccg
     1201 ccttacaacg gctctcccgc tgacgccgtc ccggactgat gggctgcctg tatcgagtgg
     1261 tgattttgtg ccgagctgcc ggtcggggag ctgttggctg gctggtggca ggatatattg
     1321 tggtgtaaac aaattgacgc ttagacaact taataacaca ttgcggacgt ttttaatgta
     1381 ctgaattaac gccgaattaa ttcgggggat ctggatttta gtactggatt ttggttttag
     1441 gaattagaaa ttttattgat agaagtattt tacaaataca aatacatact aagggtttct
     1501 tatatgctca acacatgagc gaaaccctat aggaacccta attcccttat ctgggaacta
     1561 ctcacacatt attatggaga aactcgagct acaggaacag gtggtggcgg ccctcggtgc
     1621 gctcgtactg ctccacgatg gtgtagtcct cgttgtggga ggtgatgtcc agcttggcgt
     1681 ccacgtagta gtagccgggc agctgcacgg gcttcttggc catgtagatg gacttgaact
     1741 ccaccaggta gtggccgccg tccttcagct tcagggcctt gtgggtctcg cccttcagca
     1801 cgccgtcgcg ggggtacagg cgctcggtgg aggcctccca gcccatggtc ttcttctgca
     1861 tcacggggcc gtcggagggg aagttcacgc cgatgaactt caccttgtag atgaagcagc
     1921 cgtcctgcag ggaggagtcc tgggtcacgg tcgccacgcc gccgtcctcg aagttcatca
     1981 cgcgctccca cttgaagccc tcggggaagg acagcttctt gtagtcgggg atgtcggcgg
     2041 ggtgcttcac gtacaccttg gagccgtact ggaactgggg ggacaggatg tcccaggcga
     2101 agggcagggg gccgcccttg gtcaccttca gcttcacggt gttgtggccc tcgtaggggc
     2161 ggccctcgcc ctcgccctcg atctcgaact cgtggccgtt cacggtgccc tccatgcgca
     2221 ccttgaagcg catgaactcg gtgatgacgt tctcggagga ggccataggc ccggggttct
     2281 cttctacatc acctgcctgc ttaaggaggc taaaattggt agctccgcta cctttgccga
     2341 ctaccttggt gatctcgcct ttcacgtagt ggacaaattc ttccaactga tctgcgcgcg
     2401 aggccaagcg atcttcttct tgtccaagat aagcctgtct agcttcaagt atgacgggct
     2461 gatactgggc cggcaggcgc tccattgccc agtcggcagc gacatccttc ggcgcgattt
     2521 tgccggttac tgcgctgtac caaatgcggg acaacgtaag cactacattt cgctcatcgc
```

2581 cagcccagtc gggcggcgag ttccatagcg ttaaggtttc atttagcgcc tcaaatagat
2641 cctgttcagg aaccggatca aagagttcct ccgccgctgg acctaccaag gcaacgctat
2701 gttctcttgc ttttgtcagc aagatagcca gatcaatgtc gatcgtggct ggctcgaaga
2761 tacctgcaag aatgtcattg cgctgccatt ctccaaattg cagttcgcgc ttagctggat
2821 aacgccacgg aatgatgtcg tcgtgcacaa caatggtgac ttctacagcg cggagaatct
2881 cgctctctcc aggggaagcc gaagtttcca aaaggtcgtt gatcaaagct cgccgcgttg
2941 tttcatcaag ccttacggtc accgtaacca gcaaatcaat atcactgtgt ggcttcaggc
3001 cgccatccac tgcggagccg tacaaatgta cggccagcaa cgtcggttcg agatggcgct
3061 cgatgacgcc aactacctct gatagttgag tcgatacttc ggcgatcacc gcttccccca
3121 tctcgagaga gatagatttg tagagagaga ctggtgattt cagcgtgtcc tctccaaatg
3181 aaatgaactt ccttatatag aggaagggtc ttgcgaagga tagtgggatt gtgcgtcatc
3241 ccttacgtca gtggagatat cacatcaatc cacttgcttt gaagacgtgg ttggaacgtc
3301 ttcttttttcc acgatgctcc tcgtgggtgg gggtccatct ttgggaccac tgtcggcaga
3361 ggcatcttga acgatagcct ttcctttatc gcaatgatgg catttgtagg tgccaccttc
3421 cttttctact gtccttttga tgaagtgaca gatagctggg caatggaatc cgaggaggtt
3481 tcccgatatt accctttgtt gaaaagtctc aatagccctt tggtcttctg agactgtatc
3541 tttgatattc ttggagtaga cgagagtgtc gtgctccacc atgttcacat caatccactt
3601 gctttgaaga cgtggttgga acgtcttctt tttccacgat gctcctcgtg ggtgggggtc
3661 catctttggg accactgtcg gcagaggcat cttgaacgat agcctttcct ttatcgcaat
3721 gatggcattt gtaggtgcca ccttcctttt ctactgtcct tttgatgaag tgacagatag
3781 ctgggcaatg gaatccgagg aggtttcccg atattaccct ttgttgaaaa gtctcaatag
3841 ccctttggtc ttctgagact gtatctttga tattcttgga gtagacgaga gtgtcgtgct
3901 ccaccatgtt ggcaagctgc tctagccaat acgcaaaccg cctctccccg cgcgttggcc
3961 gattcattaa tgcagctggc acgacaggtt tcccgactgg aaagcgggca gtgagcgcaa
4021 cgcaattaat gtgagttagc tcactcatta ggcaccccag gctttacact ttatgcttcc
4081 ggctcgtatg ttgtgtggaa ttgtgagcgg ataacaattt cacacaggaa acagctatga
4141 catgattacg aattcgagct caacatggtg gagcacgaca cacttgtcta ctccaaaaat
4201 atcaaagata cagtctcaga agaccaaagg gcaattgaga cttttcaaca aagggtaata
4261 tccggaaacc tcctcggatt ccattgccca gctatctgtc actttattgt gaagatagtg
4321 gaaaaggaag gtggctccta caaatgccat cattgcgata aaggaaaggc catcgttgaa
4381 gatgcctctg ccgacagtgg tcccaaagat ggacccccac ccacgaggag catcgtggaa
4441 aaagaagacg ttccaaccac gtcttcaaag caagtggatt gatgtgatat ctccactgac
4501 gtaagggatg acgcacaatc ccactatcct tcgcaagacc cttcctctat ataaggaagt
4561 tcatttcatt tggagagaac acggggactt ttacaacaat taccaacaac aacaaacaac
4621 aaacaacatt acaattacat ttacaattac catactagta tggaacaaaa gcttatctct
4681 gaggaggatc ttatggatca ttatcttgat attagactta gacctgatcc agaatttcca
4741 ccagctcaac ttatgtctgt tctttttgga aaacttcatc aagctcttgt tgctcaagga
4801 ggagatagaa ttggagtttc ttttcctgat cttgatgaat caagatcaag acttggagaa
4861 agacttagaa ttcatgcttc tgctgatgat cttagagctt tgcttgctag accttggctt
4921 gaaggactta gagatcatct tcaatttgga gaaccagctg ttgttccaca tccaactcct
4981 tatagacaag tttcaagagt tcaagctaaa tctaatccag aaagacttag aagaagactt
5041 atgagaagac atgatctttc tgaagaagaa gctagaaaaa gaattcctga tactgttgct
5101 agagctttgg atttgccttt tgttacactt agatcacaat ctactggaca acattttaga
5161 ctttttatta gacatggacc acttcaagtt actgctgaag aaggaggatt tacttgttat

5221 ggactttcta agggaggttt tgttccttgg tttggatctg gagctactaa tttttctctt
5281 cttaagcaag ctggagatgt tgaagaaaat cctggaccca tggactacaa ggacgacgat
5341 gacaaggact acaaggacga cgatgacaag acgcgtatgg ctccaaagaa gaagagaaag
5401 gttggaatcc acggagttcc agctgctgat aagaagtact ctatcggact tgctatcgga
5461 accaactctg ttggatgggc tgttatcacc gatgagtaca aggttccatc taagaagttc
5521 aaggttcttg gaaacaccga tagacactct atcaagaaga accttatcgg tgctcttctt
5581 ttcgattctg gagagaccgc tgaggctacc agattgaaga gaaccgctag aagaagatac
5641 accagaagaa agaacagaat ctgctacctt caggaaatct tctctaacga gatggctaag
5701 gttgatgatt ctttcttcca cagacttgag gagtctttcc ttgttgagga ggataagaag
5761 cacgagagac acccaatctt cggaaacatc gttgatgagg ttgcttacca cgagaagtac
5821 ccaaccatct accaccttag aaagaagttg gttgattcta ccgataaggc tgatcttaga
5881 cttatctacc ttgctcttgc tcacatgatc aagttcagag gacacttcct tatcgaggga
5941 gaccttaacc cagataactc tgatgttgat aagttgttca tccagcttgt tcagacctac
6001 aaccagcttt tcgaggagaa cccaatcaac gcttctggag ttgatgctaa ggctatcctt
6061 tctgctagac tttctaagtc tcgtagactt gagaacctta tcgctcagct tccaggagag
6121 aagaagaacg gacttttcgg aaaccttatc gctctttctc ttggacttac cccaaacttc
6181 aagtctaact tcgatcttgc tgaggatgct aagttgcagc tttctaagga tacctacgat
6241 gatgatcttg ataaccttct tgctcagatc ggagatcagt acgctgatct tttccttgct
6301 gctaagaacc tttctgatgc tatccttctt tctgacatcc ttagagttaa caccgagatc
6361 accaaggctc cactttctgc ttctatgatc aagagatacg atgagcacca ccaggatctt
6421 acccttttga aggctcttgt tagacagcag cttccagaga agtacaagga aatcttcttc
6481 gatcagtcta agaacggata cgctggatac atcgatggag gagcttctca ggaggagttc
6541 tacaagttca tcaagccaat ccttgagaag atggatggaa ccgaggagct tcttgttaag
6601 ttgaacagag aggatcttct tagaaagcag agaaccttcg ataacggatc tatcccacac
6661 cagatccacc ttggagagct tcacgctatc cttcgtagac aggaggattt ctacccattc
6721 ttgaaggata acagagagaa gatcgagaag atccttacct tcagaatccc atactacgtt
6781 ggaccacttg ctagaggaaa ctctcgtttc gcttggatga ccagaaagtc tgaggagacc
6841 atcacccctt ggaacttcga ggaggtaagt ttctgcttct acctttgata tatatataat
6901 aattatcatt aattagtagt aatataatat ttcaaatatt tttttcaaaa taaaagaatg
6961 tagtatatag caattgcttt tctgtagttt ataagtgtgt atattttaat ttataacttt
7021 tctaatatat gaccaaaatt tgttgatgtg caggttgttg ataagggagc ttctgctcag
7081 tctttcatcg agagaatgac caacttcgat aagaaccttc caaacgagaa ggttcttcca
7141 aagcactctc ttctttacga gtacttcacc gtttacaacg agcttaccaa ggttaagtac
7201 gttaccgagg gaatgagaaa gccagctttc ctttctggag agcagaagaa ggctatcgtt
7261 gatcttcttt tcaagaccaa cagaaaggtt accgttaagc agttgaagga ggattacttc
7321 aagaagatcg agtgcttcga ttctgttgaa atctctggag ttgaggatag attcaacgct
7381 tctcttggaa cctaccacga tcttttgaag atcatcaagg ataaggattt ccttgataac
7441 gaggagaacg aggacatcct tgaggacatc gttcttaccc ttaccctttt cgaggataga
7501 gagatgatcg aggagagact caagacctac gctcaccttt tcgatgataa ggttatgaag
7561 cagttgaaga gaagaagata caccggatgg ggtagacttt ctcgtaagtt gatcaacgga
7621 atcagagata agcagtctgg aaagaccatc cttgatttct tgaagtctga tggattcgct
7681 aacagaaact tcatgcagct tatccacgat gattctctta ccttcaagga ggacatccag
7741 aaggctcagg tttctggaca gggagattct cttcacgagc acatcgctaa ccttgctgga
7801 tctccagcta tcaagaaggg aatccttcag accgttaagg ttgttgatga gcttgttaag

7861 gttatgggta gacacaagcc agagaacatc gttatcgaga tggctagaga gaaccagacc
7921 acccagaagg gacagaagaa ctctcgtgag agaatgaaga gaatcgagga gggaatcaag
7981 gagcttggat ctcaaatctt gaaggagcac ccagttgaga acacccagct tcagaacgag
8041 aagttgtacc tttactacct tcagaacgga agagatatgt acgttgatca ggagcttgac
8101 atcaacagac tttctgatta cgatgttgat gctatcgttc cacagtcttt cttgaaggat
8161 gattctatcg ataacaaggt tcttacccgt tctgataaga acagaggaaa gtctgataac
8221 gttccatctg aggaggttgt taagaagatg aagaactact ggagacagct tcttaacgct
8281 aagttgatca cccagagaaa gttcgataac cttaccaagg ctgagagagg aggactttct
8341 gagcttgata aggctggatt catcaagaga cagcttgttg agaccagaca gatcaccaag
8401 cacgttgctc agatccttga ttctcgtatg aacaccaagt acgatgagaa cgataagttg
8461 atcagagagg ttaaggttat caccttgaag tctaagttgg tttctgattt cagaaaggat
8521 ttccagttct acaaggttag agagatcaac aactaccacc acgctcacga tgcttacctt
8581 aacgctgttg ttggaaccgc tcttatcaag aagtacccaa agttggagtc tgagttcgtt
8641 tacggagatt acaaggttta cgatgttaga aagatgatcg ctaagtctga gcaggagatc
8701 ggaaaggcta ccgctaagta cttcttctac tctaacatca tgaacttctt caagaccgag
8761 atcacccttg ctaacggaga gatcagaaag agaccactta tcgagaccaa cggagagacc
8821 ggagagatcg tttgggataa gggaagagat ttcgctaccg ttagaaaggt tctttctatg
8881 ccacaggtta acatcgttaa gaaaaccgag gttcagaccg gaggattctc taaggagtct
8941 atccttccaa agagaaactc tgataagttg atcgctagaa agaaggattg ggacccaaag
9001 aagtacggag gattcgattc tccaaccgtt gcttactctg ttcttgttgt tgctaaggtt
9061 gagaagggaa agtctaagaa gttgaagtct gttaaggagc ttcttggaat caccatcatg
9121 gagcgttctt ctttcgagaa gaacccaatc gatttccttg aggctaaggg atacaaggag
9181 gttaagaagg atcttatcat caagttgcca aagtactctc ttttcgagct tgagaacgga
9241 agaaagagaa tgcttgcttc tgctggagag cttcagaagg gaaacgagct tgctcttcca
9301 tctaagtacg ttaacttcct ttaccttgct tctcactacg agaagttgaa gggatctcca
9361 gaggataacg agcagaagca gcttttcgtt gagcagcaca agcactacct tgatgagatc
9421 atcgagcaaa tctctgagtt ctctaagaga gttatccttg ctgatgctaa ccttgataag
9481 gttctttctg cttacaacaa gcacagagat aagccaatca gagagcaggc tgagaacatc
9541 atccaccttt tcacccttac caaccttggt gctccagctg ctttcaagta cttcgatacc
9601 accatcgata gaaaaagata cacctctacc aaggaggttc ttgatgctac ccttatccac
9661 cagtctatca ccggacttta cgagaccaga atcgatcttt ctcagcttgg aggagataag
9721 agaccagctg ctaccaagaa ggctggacag gctaagaaga agaagcctag gggcggttcc
9781 ggctcgaaag acaatactgt gcctctgaag ctgatcgctc tcctggctaa tggcgagttc
9841 catagtggcg aacagctggg agaaaccctg ggcatgtcca gggccgctat caacaagcac
9901 attcagactc tgcgcgactg gggcgtggac gtgttcaccg tgcccggaaa gggctactct
9961 ctgcccgagc ctatcccgct gctgaacgct aaacagattc tgggacagct ggacggcggg
10021 agcgtggcag tcctgcctgt ggtcgactcc accaatcagt acctgctgga tcgaatcggc
10081 gagctgaaga gtggggatgc ttgcattgca gaatatcagc aggcagggag aggaagcaga
10141 gggaggaaat ggttctctcc ttttggagct aacctgtacc tgagtatgtt ttggcgcctg
10201 aagcggggac cagcagcaat cggcctgggc ccggtcatcg gaattgtcat ggcagaagcg
10261 ctgcgaaagc tgggagcaga caaggtgcga gtcaaatggc ccaatgacct gtatctgcag
10321 gatagaaagc tggcaggcat cctggtggag ctggccggaa taacaggcga tgctgcacag
10381 atcgtcattg gcgccgggat taacgtggct atgaggcgcg tggaggaaag cgtggtcaat
10441 cagggctgga tcacactgca ggaagcaggg attaacctgg acaggaatac tctggccgct

10501 acgctgatcc gagagctgcg ggcagccctg gaactgttcg agcaggaagg cctggctcca

10561 tatctgccac ggtgggagaa gctggataac ttcatcaata gacccgtgaa gctgatcatt

10621 ggggacaaag agattttcgg gattagccgg gggattgata aacagggagc cctgctgctg

10681 gaacaggacg gagttatcaa accctggatg ggcggagaaa tcagtctgcg gtctgccgaa

10741 aagtgaccta ggggatcata tgaagatgaa gatgaaatat ttggtgtgtc aaataaaaag

10801 gttgtgtgct taagtttgtg tttttttctt ggcttgttgt gttatgaatt tgtggctttt

10861 tctaatatta aatgaatgta acatctcatt ataatgaata aacaaatgtt tctataatcc

10921 attgtgaatg ttttgttgga tctcttctcc agcatataac tactgtatgt gctatggtat

10981 ggactatgga atatgattaa agataaggga tccaagcttt ctccataata atgtgtgagt

11041 agttcccaga taagggaatt agggttccta tagggtttcg ctcatgtgtt gagcatataa

11101 gaaaccctta gtatgtattt gtatttgtaa aatacttcta tcaataaaat ttctaattcc

11161 taaaaccaaa atccagtact aaaatccaga tccccgtcga cctgcaggca tgcaagcttg

11221 gcactggccg tcgttttaca acgtcgtgac tgggaaaacc ctggcgttac ccaacttaat

11281 cgccttgcag cacatccccc tttcgccagc tggcgtaata gcgaagaggc ccgcaccgat

11341 cgcccttccc aacagttgcg cagcctgaat ggcgaatgct agagcagctt gagcttggat

11401 cagattgtcg tttcccgcct tcagtttaaa ctatcagtgt ttgacaggat atattggcgg

11461 gtaaacctaa gagaaaagag cgtttattag aataatcgga tatttaaaag ggcgtgaaaa

11521 ggtttatccg ttcgtccatt tgtatgtgca tgccaaccac agggttcccc tcgggatcaa

11581 agtactttga tccaacccct ccgctgctat agtgcagtcg gcttctgacg ttcagtgcag

11641 ccgtcttctg aaaacgacat gtcgcacaag tcctaagtta cgcgacaggc tgccgccctg

11701 cccttttcct ggcgttttct tgtcgcgtgt tttagtcgca taaagtagaa tacttgcgac

11761 tagaaccgga gacattacgc catgaacaag agcgccgccg ctggcctgct gggctatgcc

11821 cgcgtcagca ccgacgacca ggacttgacc aaccaacggg ccgaactgca cgcggccggc

11881 tgcaccaagc tgttttccga gaagatcacc ggcaccaggc gcgaccgccc ggagctggcc

11941 aggatgcttg accacctacg ccctggcgac gttgtgacag tgaccaggct agaccgcctg

12001 gcccgcagca cccgcgacct actggacatt gccgagcgca tccaggaggc cggcgcgggc

12061 ctgcgtagcc tggcagagcc gtgggccgac accaccacgc cggccggccg catggtgttg

12121 accgtgttcg ccggcattgc cgagttcgag cgttccctaa tcatcgaccg cacccggagc

12181 gggcgcgagg ccgccaaggc ccgaggcgtg aagtttggcc cccgccctac cctcaccccg

12241 gcacagatcg cgcacgcccg cgagctgatc gaccaggaag gccgcaccgt gaaagaggcg

12301 gctgcactgc ttggcgtgca tcgctcgacc ctgtaccgcg cacttgagcg cagcgaggaa

12361 gtgacgccca ccgaggccag gcggcgcggt gccttccgtg aggacgcatt gaccgaggcc

12421 gacgccctgg cggccgccga gaatgaacgc caagaggaac aagcatgaaa ccgcaccagg

12481 acggccagga cgaaccgttt ttcattaccg aagagatcga ggcggagatg atcgcggccg

12541 ggtacgtgtt cgagccgccc gcgcacgtct caaccgtgcg gctgcatgaa atcctggccg

12601 gtttgtctga tgccaagctg gcggcctggc cggccagctt ggccgctgaa gaaaccgagc

12661 gccgccgtct aaaaaggtga tgtgtatttg agtaaaacag cttgcgtcat gcggtcgctg

12721 cgtatatgat gcgatgagta aataaacaaa tacgcaaggg gaacgcatga aggttatcgc

12781 tgtacttaac cagaaaggcg ggtcaggcaa gacgaccatc gcaacccatc tagcccgcgc

12841 cctgcaactc gccggggccg atgttctgtt agtcgattcc gatccccagg gcagtgcccg

12901 cgattgggcg gccgtgcggg aagatcaacc gctaaccgtt gtcggcatcg accgcccgac

12961 gattgaccgc gacgtgaagg ccatcggccg gcgcgacttc gtagtgatcg acggagcgcc

13021 ccaggcggcg gacttggctg tgtccgcgat caaggcagcc gacttcgtgc tgattccggt

13081 gcagccaagc ccttacgaca tatgggccac cgccgacctg gtggagctgg ttaagcagcg

13141 cattgaggtc acggatggaa ggctacaagc ggcctttgtc gtgtcgcggg cgatcaaagg

13201 cacgcgcatc ggcggtgagg ttgccgaggc gctggccggg tacgagctgc ccattcttga

13261 gtcccgtatc acgcagcgcg tgagctaccc aggcactgcc gccgccggca caaccgttct

13321 tgaatcagaa cccgagggcg acgctgcccg cgaggtccag gcgctggccg ctgaaattaa

13381 atcaaaactc atttgagtta atgaggtaaa gagaaaatga gcaaaagcac aaacacgcta

13441 agtgccggcc gtccgagcgc acgcagcagc aaggctgcaa cgttggccag cctggcagac

13501 acgccagcca tgaagcgggt caactttcag ttgccggcgg aggatcacac caagctgaag

13561 atgtacgcgg tacgccaagg caagaccatt accgagctgc tatctgaata catcgcgcag

13621 ctaccagagt aaatgagcaa atgaataaat gagtagatga attttagcgg ctaaaggagg

13681 cggcatggaa aatcaagaac aaccaggcac cgacgccgtg gaatgcccca tgtgtggagg

13741 aacgggcggt tggccaggcg taagcggctg ggttgcctgc cggccctgca atggcactgg

13801 aacccccaag cccgaggaat cggcgtgagc ggtcgcaaac catccggccc ggtacaaatc

13861 ggcgcggcgc tgggtgatga cctggtggag aagttgaagg ccgcgcaggc cgcccagcgg

13921 caacgcatcg aggcagaagc acgccccggt gaatcgtggc aagcggccgc tgatcgaatc

13981 cgcaaagaat cccggcaacc gccggcagcc ggtgcgccgt cgattaggaa gccgcccaag

14041 ggcgacgagc aaccagattt tttcgttccg atgctctatg acgtgggcac ccgcgatagt

14101 cgcagcatca tggacgtggc cgttttccgt ctgtcgaagc gtgaccgacg agctggcgag

14161 gtgatccgct acgagcttcc agacgggcac gtagaggttt ccgcagggcc ggccggcatg

14221 gccagtgtgt gggattacga cctggtactg atggcggttt cccatctaac cgaatccatg

14281 aaccgatacc gggaagggaa gggagacaag cccggccgcg tgttccgtcc acacgttgcg

14341 gacgtactca agttctgccg gcgagccgat ggcggaaagc agaaagacga cctggtagaa

14401 acctgcattc ggttaaacac cacgcacgtt gccatgcagc gtacgaagaa ggccaagaac

14461 ggccgcctgg tgacggtatc cgagggtgaa gccttgatta gccgctacaa gatcgtaaag

14521 agcgaaaccg ggcggccgga gtacatcgag atcgagctag ctgattggat gtaccgcgag

14581 atcacagaag gcaagaaccc ggacgtgctg acggttcacc ccgattactt tttgatcgat

14641 cccggcatcg gccgttttct ctaccgcctg gcacgccgcg ccgcaggcaa ggcagaagcc

14701 agatggttgt tcaagacgat ctacgaacgc agtggcagcg ccggagagtt caagaagttc

14761 tgtttcaccg tgcgcaagct gatcgggtca aatgacctgc cggagtacga tttgaaggag

14821 gaggcggggc aggctggccc gatcctagtc atgcgctacc gcaacctgat cgagggcgaa

14881 gcatccgccg gttcctaatg tacggagcag atgctagggc aaattgccct agcaggggaa

14941 aaaggtcgaa aaggtctctt tcctgtggat agcacgtaca ttgggaaccc aaagccgtac

15001 attgggaacc ggaacccgta cattgggaac ccaaagccgt acattgggaa ccggtcacac

15061 atgtaagtga ctgatataaa agagaaaaaa ggcgattttt ccgcctaaaa ctctttaaaa

15121 cttattaaaa ctcttaaaac ccgcctggcc tgtgcataac tgtctggcca gcgcacagcc

15181 gaagagctgc aaaaagcgcc taccсttcgg tcgctgcgct ccctacgccc cgccgcttcg

15241 cgtcggccta tcgcggccgc tggccgctca aaaatggctg gcctacggcc aggcaatcta

15301 ccagggcgcg gacaagccgc gccgtcgcca ctcgaccgcc ggcgcccaca tcaaggcacc

15361 ctgcctcgcg cgtttcggtg atgacggtga aaacctctga cacatgcagc tcccggagac

15421 ggtcacagct tgtctgtaag cggatgccgg gagcagacaa gcccgtcagg gcgcgtcagc

15481 gggtgttggc gggtgtcggg gcgcagccat gacccagtca cgtagcgata gcggagtgta

15541 tactggctta actatgcggc atcagagcag attgtactga gagtgcacca tatgcggtgt

15601 gaaataccgc acagatgcgt aaggagaaaa taccgcatca ggcgctcttc cgcttcctcg

15661 ctcactgact cgctgcgctc ggtcgttcgg ctgcggcgag cggtatcagc tcactcaaag

15721 gcggtaatac ggttatccac agaatcaggg gataacgcag gaaagaacat gtgagcaaaa

15781 ggccagcaaa aggccaggaa ccgtaaaaag gccgcgttgc tggcgttttt ccataggctc
15841 cgcccccctg acgagcatca caaaaatcga cgctcaagtc agaggtggcg aaacccgaca
15901 ggactataaa gataccaggc gtttccccct ggaagctccc tcgtgcgctc tcctgttccg
15961 accctgccgc ttaccggata cctgtccgcc tttctccctt cgggaagcgt ggcgctttct
16021 catagctcac gctgtaggta tctcagttcg gtgtaggtcg ttcgctccaa gctgggctgt
16081 gtgcacgaac cccccgttca gcccgaccgc tgcgccttat ccggtaacta tcgtcttgag
16141 tccaacccgg taagacacga cttatcgcca ctggcagcag ccactggtaa caggattagc
16201 agagcgaggt atgtaggcgg tgctacagag ttcttgaagt ggtggcctaa ctacggctac
16261 actagaagga cagtatttgg tatctgcgct ctgctgaagc cagttacctt cggaaaaaga
16321 gttggtagct cttgatccgg caaacaaacc accgctggta gcggtggttt ttttgtttgc
16381 aagcagcaga ttacgcgcag aaaaaaagga tctcaa

Figure S1. Map and full sequence of dTAPL plasmid.

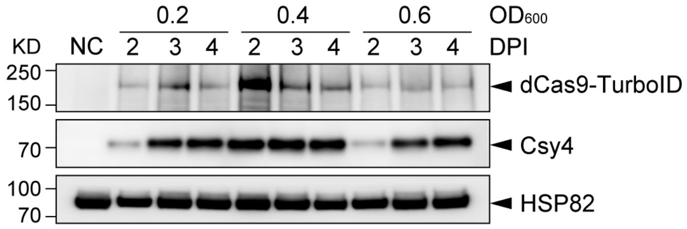

Figure S2. Optimization of experimental conditions used for dTAPL in tobacco. Immunoblot assay showing the expression levels of indicated proteins in tobacco leaves. NC, negative control (agrobacterium without dTAPL plasmids is used for leaf infiltration), DPI, days post infiltration.

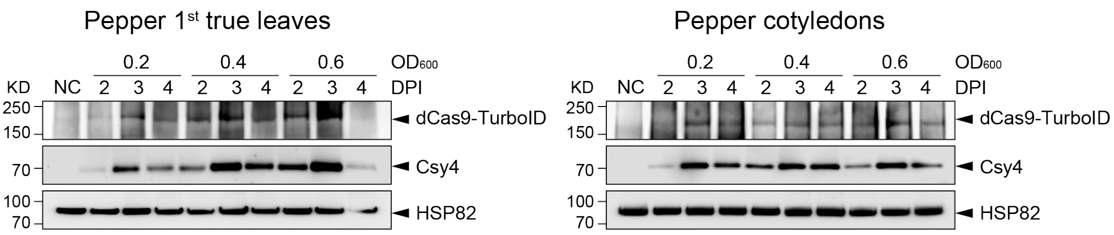

Figure S3. Examination of dTAPL expressions in pepper seedlings (*Capsicum annuum*) by agroinfiltration. Immunoblot assay showing the expression levels of indicated proteins in pepper cotyledons and leaves. NC, negative control (agrobacterium without dTAPL plasmids is used for leaf infiltration), DPI, days post infiltration.

Chromosome	Start	End	Gene ID	Annotation
Chr01	7322338	7322545	Nbe01g02850	Exon
Chr01	31725693	31726063	Nbe01g11060	Promoter (<=1kb)
Chr01	61024337	61024574	Nbe01g20000	Distal Intergenic
Chr01	64364894	64365047	Nbe01g20910	Exon
Chr02	33572551	33572692	Nbe02g10050	3'UTR
Chr02	103699556	103699805	Nbe02g28180	Promoter (<=1kb)
Chr03	22339129	22339289	Nbe03g04270	Distal Intergenic
Chr03	23860168	23860438	Nbe03g04690	Distal Intergenic
Chr03	36802288	36802482	Nbe03g07580	Distal Intergenic
Chr03	37809197	37809564	Nbe03g07690	Distal Intergenic
Chr03	46246988	46247193	Nbe03g09050	Distal Intergenic
Chr03	87394759	87394929	Nbe03g17650	Distal Intergenic
Chr03	97428370	97428533	Nbe03g20910	Distal Intergenic
Chr03	132099043	132099258	Nbe03g33540	Distal Intergenic
Chr04	45789673	45789986	Nbe04g11940	Distal Intergenic
Chr04	59534417	59534601	Nbe04g15460	Distal Intergenic
Chr04	97803994	97804146	Nbe04g24690	Distal Intergenic
Chr05	28881542	28881758	Nbe05g06160	Distal Intergenic
Chr05	45587877	45588065	Nbe05g09680	Distal Intergenic
Chr05	86446401	86446654	Nbe05g18100	Distal Intergenic
Chr05	92702259	92702480	Nbe05g20080	Promoter (<=1kb)
Chr05	131941166	131941520	Nbe05g35010(PDS)	Promoter (<=1kb)
Chr05	131941927	131942732	Nbe05g35010(PDS)	Promoter (<=1kb)
Chr05	131942299	131942735	Nbe05g35010(PDS)	Promoter (<=1kb)
Chr06	7236150	7236362	Nbe06g02180	Intron
Chr06	48630476	48630641	Nbe06g13170	Distal Intergenic
Chr06	66172836	66172961	Nbe06g17070	Distal Intergenic
Chr06	100164418	100164680	Nbe06g28420	Distal Intergenic
Chr06	104244120	104244430	Nbe06g29850	Distal Intergenic
Chr06	113499878	113500081	Nbe06g32540	Promoter (<=1kb)
Chr06	130994106	130994257	Nbe06g36450	Promoter (<=1kb)
Chr08	27245313	27245464	Nbe08g06390	Distal Intergenic
Chr08	38346490	38346708	Nbe08g09540	Distal Intergenic
Chr08	58271007	58271167	Nbe08g14420	Distal Intergenic
Chr09	7822913	7823119	Nbe09g02350	Distal Intergenic
Chr09	86667440	86667565	Nbe09g20900	Distal Intergenic
Chr09	98122589	98122745	Nbe09g23960	Distal Intergenic
Chr10	23680247	23680481	Nbe10g05760	Distal Intergenic
Chr10	44803538	44803706	Nbe10g10620	Distal Intergenic
Chr10	46057507	46057662	Nbe10g10950	Distal Intergenic
Chr10	46102546	46102924	Nbe10g10960	Distal Intergenic
Chr10	46123635	46123845	Nbe10g10980	Promoter (1-2kb)
Chr10	46198969	46199181	Nbe10g11050	Distal Intergenic
Chr11	17516254	17516468	Nbe11g03990	Distal Intergenic
Chr11	66727455	66727612	Nbe11g12660	Promoter (<=1kb)
Chr12	85865190	85865360	Nbe12g17470	Distal Intergenic
Chr12	120963645	120963777	Nbe12g25090	Distal Intergenic
Chr13	5560762	5560996	Nbe13g01070	Distal Intergenic
Chr13	6524887	6525072	Nbe13g01340	Distal Intergenic
Chr13	111054119	111054291	Nbe13g23860	Distal Intergenic
Chr14	30133657	30133819	Nbe14g07670	Distal Intergenic
Chr14	70017511	70017708	Nbe14g16950	Distal Intergenic
Chr15	20923602	20923758	Nbe15g05330	Distal Intergenic
Chr15	67202434	67202674	Nbe15g16700	Distal Intergenic
Chr16	73886967	73887194	Nbe16g15410	Distal Intergenic
Chr16	114407598	114407783	Nbe16g25290	Promoter (1-2kb)
Chr17	2154317	2154566	Nbe17g00930	Distal Intergenic
Chr17	14727213	14727465	Nbe17g04150	Promoter (<=1kb)
Chr17	14789713	14789899	Nbe17g04160	Promoter (<=1kb)
Chr17	67240051	67240263	Nbe17g14980	Distal Intergenic
Chr17	87494877	87495032	Nbe17g19390	Promoter (<=1kb)
Chr19	59598085	59598332	Nbe19g13770	Distal Intergenic
Chr19	107618749	107618919	Nbe19g23020	Distal Intergenic

Figure S4. Genome-wide binding peaks of dTAPL-*gRNA*^{PDS} in ChIP-seq assay.

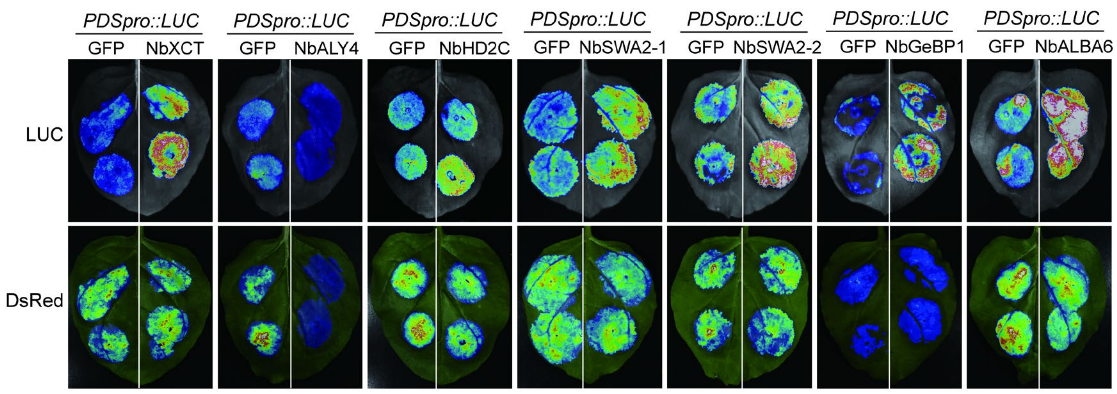

Figure S5. Tobacco leaf images of LUC reporter assays.

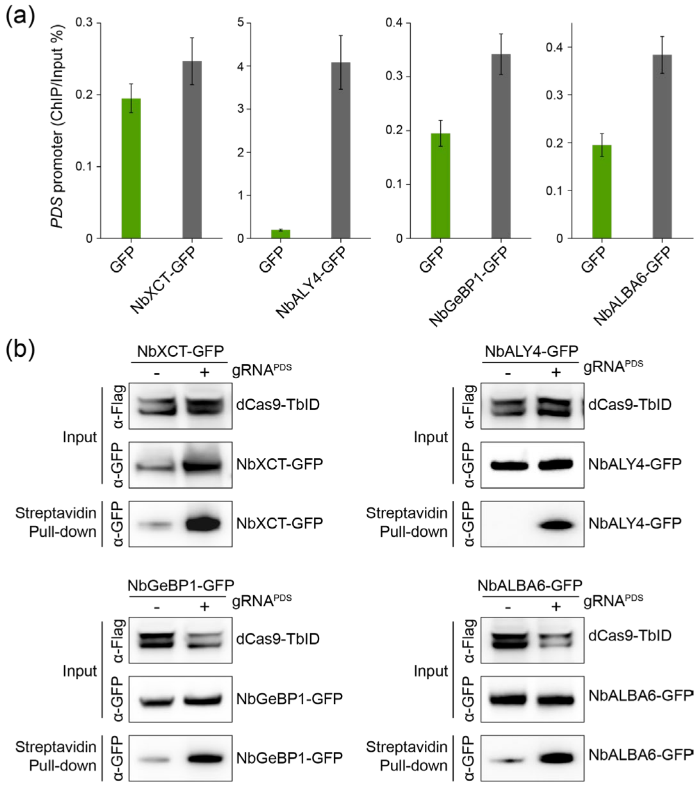

Figure S6. Validation of the results obtained by dTAPL-*gRNA^{PDS}*. (a) ChIP-qPCR results showing that NbXCT-GFP, NbALY4-GFP, NbGeBP1-GFP and NbALBA6-GFP proteins associate with *PDS* promoter *in vivo*. Indicated proteins were transiently expressed in tobacco leaves by agroinfiltration. Then the leaf tissues were used for ChIP assay. (b) Proximity labeling assays showing that NbXCT-GFP, NbALY4-GFP, NbGeBP1-GFP and NbALBA6-GFP proteins could be more efficiently labeled by dCas9-TurboID in the presence of gRNA^{PDS}. dTAPL-gRNA^{PDS} or dTAPL (without gRNA) with indicated proteins were co-expressed in tobacco leaves. Then the leaves were fed with biotin and subjected to protein extraction and streptavidin-pull down. The indicated proteins in both input and pull-down samples were detected by immunoblots.

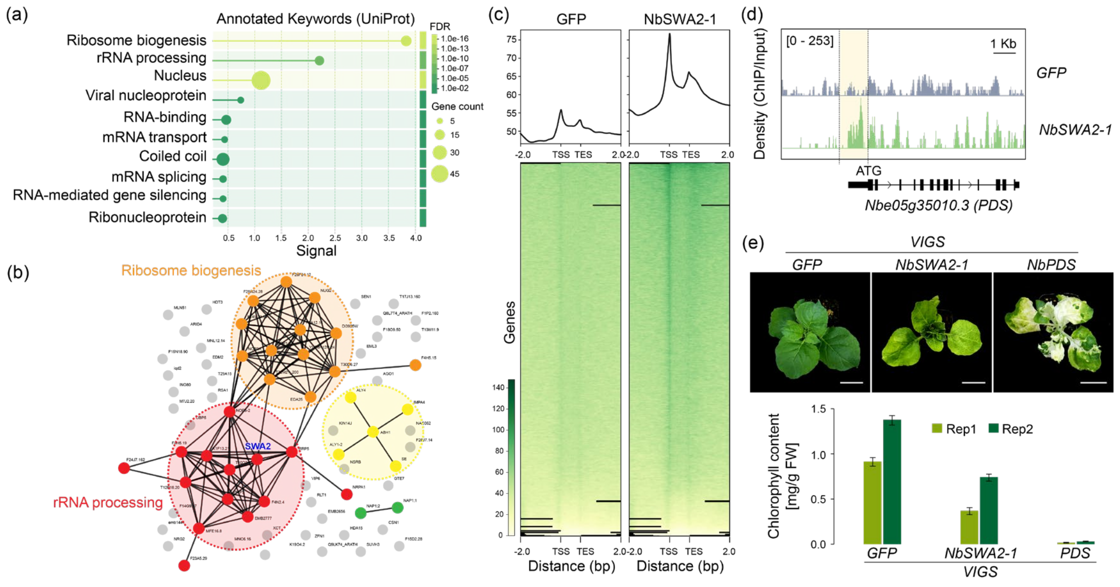

Figure S7. Evidence suggesting that rRNA-related factors may play a role in regulating *PDS* gene expression in tobacco. (a) Enrichment of annotated key words of identified candidate proteins bound with *PDS* promoter by dTAPL, which are predicted as nuclear proteins. (b) Construction of protein-protein interaction (PPI) network with the same group of proteins in (a). (c) Metaplots and heatmaps of NbSWA2-1 ChIP-seq reads over genes. GFP and NbSWA2-1-GFP are independently expressed in tobacco leaves by agroinfiltration which are used for ChIP-seq assays. GFP is used as the negative control. TSS, transcription start site; TES, transcription end site; −2.0, 2 kb upstream of TSS; 2.0, 2 kb downstream of TES. (d) Snapshot of NbSWA2-1 binding to *PDS* promoter. (e) VIGS assay in tobacco showing that silencing of *NbSWA2-1* gene led to significant reduction of total chlorophyll contents in leaves. *PDS* and *GFP* are used as positive and negative controls for VIGS assay, respectively. Scale bar= 5 cm.

Materials and Methods

Plant growth conditions

N. benthamiana plants were grown under in a climate-controlled chamber with 16 h light/8 h dark photoperiod at 22°C.

Construction of plasmids

The primers used for the construction of plasmids described below are listed in Supplementary Table S2 and DNA sequencing was performed to confirm all the plasmids. dCas9, TurboID and HSP terminator fragments were generated by PCR using the HBT-35Spro-dCas9-TV (Li et al., 2017), 35S-YFP-TbID (from our lab) and pDIRECT_23C (Addgene #91130) plasmids as templates, and the resulting fragments were cloned into 35S::2×Flag-GFP plasmid using MluI/BamHI, AvrII, and BamHI restriction sites respectively to generate the 35S::2×Flag-dCas9-TurboID-HSPter. Then, the PCR amplified Myc-Csy4-P2A-2×FLAG fragment using pDIRECT_23C as a template was cloned into SpeI/MluI digested 35S::2×Flag-dCas9-TurboID-HSPter vector to construct dTAPL plasmid.
For proof-of-concept testing of dTAPL, three gRNAS were designed in PDS promoter region and assembled into pDIRECT_23C (Čermák et al., 2017). Then, the CmYLCYpro-Csy4-sgRNA-gRNA scaffold fragment was amplified by PCR and the resulting fragment was ligated into BamHI digested dTAPL construct.
For the subcellular localization and LUC reporter assays, coding region of the candidates were PCR amplified and cloned into 35S::2×Flag-GFP linearized with SpeI/XmaI. PDSpro::LUC-UBQ10::DsRed plasmid was constructed by replacing the ACT2pro in the plasmid ACT2pro::LUC-UBQ10::DsRed with PCR amplified PDSpro fragment using SacI/SpeI restriction sites.

TurboID sample preparation for MS analysis

The dTAPL construct were transformed into Agrobacterium tumefaciens strain GV3101(P19) for the infiltration of four-week-old N. benthamiana leaves. The bacterial suspension was diluted with supplemented infiltration buffer [10mM MES (pH5.6), 10mM MgCl2, and 100uM Acetosyringone] to adjust the inoculum concentration to an OD600 value of 0.2 to 0.6.
48h later, infiltration solution containing 0-200 um biotin was infiltrated into the same leaf tissues. Three infiltrated leaves from each plant were then harvested after 12h incubation and frozen in liquid nitrogen. The finely ground frozen leaf material was resuspended in an equal volume of ice-cold protein extraction buffer [50 mM Tris (pH7.5), 150 mM NaCl, 0.5% (w/v) Sodium deoxycholate, 0.1% SDS (w/v), 1 mM EDTA, 1% Triton X-100, 1 mM DTT, 1 mM PMSF, 1×Protease Inhibitor Cocktail] and incubated at 4°C for 1 h on a rotor wheel. Then, the lysates were cleared by centrifugation for 15 min at 14000 rpm (4°C), and the supernatant was used directly or filtered through ZebaTM Spin Desalting Columns (Thermo Fisher Scientific) to remove the excess biotin in lysates.
To enrich biotinylated proteins, the desalted lysates were then incubated with the equilibrated streptavidin-coated magnetic beads (DynabeadsTM MyOneTM

streptavidin C1, Invitrogen) on a rotator overnight at 4°C. The beads were sequentially washed twice with protein extraction buffer, once with 1 M KCl, once with 100 mM Na2CO3, once with 2 M urea in 10 mM Tris-HCl (pH 8.0), twice with protein extraction buffer. To confirm the successful enrichment of the biotinylated proteins, two percent of the suspension was taken out for Western blot analysis and the rest of the beads were stored at -80°C or sent immediately on the dry ice for LC-MS/MS analysis.

LC-MS/MS analysis

Biotinylated proteins enriched with streptavidin beads were processed into peptides via on bead digestion and analyzed by LC-MS/MS according to the previously described methods (Qin et al., 2017). The raw files were processed using Proteome Discoverer software (Thermo Fisher Scientific, version 2.4) for peptide identification and searched against the *N. benthamiana* reference proteome (Wang et al., 2024). The following parameters were used: oxidation of Met was set as variable modifications, and a maximum of two missed cleavages was allowed. The false discovery rates of the peptides were set at 1% FDR.

ChIP-seq and data processing

The ChIP assay was performed as previously reported (Yang et al., 2025). Specifically, the tobacco chromatin was sheared with Bioruptor Plus (Diagenode) for 30 cycles with 30 s ON/30 s OFF and incubated with anti-flag (Sigma-Aldrich) overnight at 4°C. Two biological replicates were performed for each ChIP, and libraries were prepared with VAHTS Universal DNA Library Prep Kit for Illumina V4 (Vazyme, Cat. ND610) and VAHTS Multiplex Oligos Set 4 for Illumina (Vazyme, Cat. N321) kits following the manufacturer's instructions.

All libraries were sequenced at a length of 150 bp pair-end with NovaSeq 6000 platform (Illumina). The raw paired-end reads were trimmed to remove adaptors and low-quality bases using Fastp (v.0.23.4). The reads were filtered with a sliding window of size 5, with an average Phred score of 20 within the window and reads containing more than 5 N bases were also removed. All clean reads were aligned to the N. benthamiana reference genome (Wang et al., 2024) using the 'MEM' algorithm in the Burrows-Wheeler Aligner (bwa-mem v0.7.17-r1188). Peaks were called with MACS2 (v2.1.1) (q value < 0.01) (Zhang et al., 2008).

qRT-PCR assay

Total RNA was isolated using TRIzol reagent (Invitrogen). First-strand cDNA was synthesized according to the standard protocol of the TransScript® one-step gDNA removal and cDNA synthesis super mix kit (TransGen Biotech). The synthetic cDNA was used as a template for qRT-PCR performed with 2×SYBR Green Mix (GeneStar) on a CFX Opus 96 instrument (Bio-Rad). Three replicates were carried out for each sample. Expression levels of PDS were normalized using the CaActin gene. The primers used for qRT-PCR analysis are listed in Supplementary Table S2.

Luciferase reporter assay

PDSpro::LUC-UBQ10::DsRed plasmid was a reporter construct, and 35S::NbXCT-GFP, 35S::NbALY4-GFP, 35S::NbHD2C-GFP, 35S::NbSWA2-1-GFP, 35S::NbSWA2-2-GFP, 35S::NbGeBP1-GFP, and 35S::NbALBA6-GFP worked as reporter constructs. These constructs were transformed into A. tumefaciens strain GV3101(P19). In brief, bacterial cells were centrifuged at 4000 g for 10 min and resuspended in infiltration buffer [10 mM MES (pH5.6), 10 mM MgCl2, and 100 uM Acetosyringone] with OD600 value of 0.7-0.8. The reporter was mixed with the different effectors in the ratio of 1:1 (v/v) and incubated for 3 h before infiltration into tobacco leaves for expression. After three days, the leaves were photoed by Lumazone PyLoN 2048B (Lumazone) and Amersham ImageQuant 800 Fluor (Cytiva), respectively. Image analyses were performed using ImageJ (Schindelin et al., 2012), then values of luciferase activity were calculated by normalizing to the values of DsRed protein level. The assay was repeated more than five times independently.

Subcellular localization

35S::NbXCT-GFP, 35S::NbALY4-GFP, 35S::NbHD2C-GFP, 35S::NbSWA2-1-GFP, 35S::NbSWA2-2-GFP, 35S::NbGeBP1-GFP, and 35S::NbALBA6-GFP were co-infiltrated in tobacco leaves with H2B-BFP, respectively. A laser-scanning confocal microscope (Nikon ECLIPSE Ti2) was used to detect the fluorescent signals between 48 h and 72 h after co-injection (Lasers: GFP: 488 nm, BFP: 405 nm).

Reference

Čermák, T., Curtin, S.J., Gil-Humanes, J., Čegan, R., Kono, T.J.Y., Konečná, E., Belanto, J.J., Starker, C.G., Mathre, J.W., Greenstein, R.L. and Voytas, D.F. (2017) A Multipurpose Toolkit to Enable Advanced Genome Engineering in Plants. Plant Cell 29, 1196-1217.

Chen, S.F., Zhou, Y.Q., Chen, Y. and Gu, J. fastp: an ultra-fast all-in-one FASTQ preprocessor. (2018) Bioinformatics 34, i884-i890.

Li, H. and Durbin, R. Fast and accurate short read alignment with Burrows-Wheeler transform. (2009) Bioinformatics 25, 1754-1760.

Li, Z., Zhang, D.D., Xiong, X.Y., Yan, B.Y., Xie, W., Sheen, J. and Li, J.-F. (2017) A potent Cas9-derived gene activator for plant and mammalian cells. Nature Plants 3, 930-936.

Qin, G.C., Ma, J., Chen, X.M., Chu, Z.Q. and She, Y.-M. (2017) Methylated-antibody affinity purification to improve proteomic identification of plant RNA polymerase Pol V complex and the interacting proteins. Scientific Reports 7, 42943.

Schindelin, J., Arganda-Carreras, I., Frise, E., Kaynig, V., Longair, M., Pietzsch, T., Preibisch, S., Rueden, C., Saalfeld, S., Schmid, B., Tinevez, J.-Y., White, D.J., Hartenstein, V., Eliceiri, K., Tomancak, P. and Cardona, A. (2012) Fiji: an open-source platform for biological-image analysis. Nature Methodes 28, 676-682.

Wang, J., Zhang, Q.L., Tung, J., Zhang, X., Liu, D., Deng, Y.T., Tian, Z.D., Chen, H., Wang, T.T., Yin, W.X., Li, B., Lai, Z.B., Dinesh-Kumar, S.P., Baker, B. and Li, F. (2024) High-quality assembled and annotated genomes of Nicotiana tabacum and Nicotiana benthamiana reveal chromosome evolution and changes in defense arsenals. Molecular

Plant 17, 423-437.
Yang, H.J., Yu, G.R., Lv, Z.D., Li, T.H., Wang, X., Fu, Y., Zhu, Z.S., Guo, G.J., He, H., Wang, M., Qin, G.C., Liu, F., Zhong, Z.H. and Xue, Y. (2025) Epigenome profiling reveals distinctive regulatory features and cis-regulatory elements in pepper. Genome Biology 26, 121.
Zhang, Y., Liu, T., Meyer, C.A., Eeckhoute, J., Johnson, D.S., Bernstein, B.E., Nusbaum, C., Myers, R.M., Brown, M., Li, W. and Liu, X.S. (2008) Model-based analysis of ChIP-Seq (MACS). Genome Biology 9, R137.
